# Effects of fire on ground‐dwelling arthropods in a shrub‐dominated grassland

**DOI:** 10.1002/ece3.7063

**Published:** 2020-12-07

**Authors:** Anna Butler, Craig A. Davis, Samuel D. Fuhlendorf, Shawn M. Wilder

**Affiliations:** ^1^ Department of Integrative Biology Oklahoma State University Stillwater OK USA; ^2^ Department of Natural Resource Ecology and Management Oklahoma State University Stillwater OK USA

**Keywords:** arthropods, grassland, mottes, prescribed burning

## Abstract

Arthropods are abundant and diverse animals in many terrestrial food webs. In western Oklahoma, some shrublands are interspersed with discrete, dense thickets of tall, woody vegetation, known as mottes. Some of these shrublands are managed with prescribed burning. The goal of this study was to examine whether prescribed burning interacted with habitat type (i.e., shrubland versus mottes) to affect ground‐dwelling arthropod communities. Arthropods were collected in pitfall traps at four sampling locations in relation to mottes; in the center of mottes, and three plot location in shrublands; 1 m, 15 m, and 50 m away from the edge of the motte. There were three treatment levels for burning: one year postburn (burned in dormant months of 2017), two years postburn (burned in dormant months of 2016), and unburned (burned in dormant season of 2014 and prior). There were no significant interactions between prescribed burning and habitat type. Mottes had a different community of arthropods compared with the surrounding shrubland. Mottes also had lower overall abundance, but a higher diversity of arthropods. In terms of fires, arthropod communities one year after burning were different from those two or more years after burning. There was no effect of burning on overall arthropod abundance, but plots that were one year since burning had significantly lower diversity compared with plots that were two or more years postburn. The results of this study suggest that both fire and mottes can independently facilitate heterogeneity in arthropod communities, but they do not appear to interact with one another.

## Graphical abstract

The goal of this study was to examine whether prescribed burning interacted with habitat type (i.e., shrubland versus mottes) to affect ground‐dwelling arthropod communities. The results of this study suggest that both fire and mottes can independently facilitate heterogeneity in arthropod communities, but they do not appear to interact with one another.

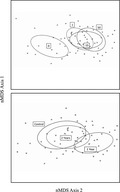

## 1 INTRODUCTION

Arthropods are an abundant and diverse group of invertebrates in many terrestrial food webs. They have important roles as decomposers that facilitate nutrient cycling, herbivores that can alter vegetation structure and composition, pollinators, and food for larger animals such as vertebrates (Seastedt & Crossley, 1984). Yet, recent work has found that 41% of insect species are in decline and about one third of all insect species are threatened (Sánchez‐Bayo & Wyckhuys, 2019), highlighting the vulnerability of invertebrates to climate change and other anthropogenic impacts (Deutsch et al., 2008; Gillespie et al., 2020; Hallmann et al., 2017; Lister & Garcia, 2018). In addition, estimates suggest that the overall biomass of arthropods is decreasing at an annual rate of 2.5% worldwide (Sánchez‐Bayo & Wyckhuys, 2019). These declines in abundance and diversity of arthropods are alarming given their importance for food web and ecosystem function (Klink et al., 2020; Wagner, 2020). Understanding the factors that influence the abundance and biomass of arthropods is critical for managing their populations and maintaining the ecosystem services that they provide.

Vegetation structure and composition are known to be important factors affecting arthropod abundance and diversity (Prather & Kaspari, 2019). In sandy soils in western Oklahoma, the prairie can consist of grassland interspersed with shinnery oak shrubs (*Quercus havardii*) and mottes, which are dense patches of taller (e.g., >2 m tall) oak trees (Peterson & Boyd, 2000; Weideman & Penfound, 1960). Mottes are unique in this landscape because they form small patches of trees dispersed within the shrublands. The vegetation structure provided by mottes can create thermal refugia to allow some species, including northern bobwhite (*Colinus virginianus*) and wild turkey (*Meleagris gallopavo*), to escape high temperatures experienced in the relatively open shrubland habitat (Carroll et al., 2015; Rakowski et al., 2019). For example, female northern bobwhite with broods will often use mottes and other areas of shade during the middle of the day to reduce the temperatures they experience by up to 10°C relative to other locations in the landscape (Carroll et al., 2015). Thermoregulation is also important for ectotherms, such as arthropods (May, 1979), although less is known of the response of arthropods to mottes. Mottes may support different arthropod communities relative to the surrounding shrubland, due to the different microclimates, and mottes may provide areas for more wide‐ranging arthropods to avoid mid‐day temperature extremes (Robertson et al., 1996).

Prescribed fire is a management tool that has historically been used in prairie ecosystems in the Great Plains to reduce woody plant encroachment. Prescribed fire has also been used to restore heterogeneity in grasslands by creating a shifting mosaic of different seral stages of vegetation across the landscape through the interaction of fire and grazing (Fuhlendorf et al., 2009). The ecological interaction between fire and grazing, known as pyric herbivory, can reduce the abundance of woody vegetation, change vegetation quality, and shift the successional stage of vegetation (Fuhlendorf & Engle, 2004).

Changes in vegetation, in turn, can affect invertebrate communities since invertebrate communities are often dependent on plant community composition and structure (Engle et al., 2008; Reed, 1997). In grassland and shrubland ecosystems, arthropod taxa are differentially affected by prescribed burning depending on their life stage during the burn, habitat use (e.g., aboveground versus belowground), dispersal ability, and other factors (Swengel, 2001; Warren et al., 1987). Arthropod communities can also shift with time since fire due to successional changes in plant communities and accumulation of litter (Swengel, 2001; Warren et al., 1987). By burning different patches at different times, insect diversity and abundance can be increased across landscapes (Doxon et al., 2011; Fuhlendorf & Engle, 2004; Fuhlendorf et al., 2006). However, while fire has been studied in a number of grassland systems, it remains less clear how arthropod communities in grasslands interspersed with shrubs and mottes respond to prescribed fire. In particular, arthropod communities in mottes may respond differently than arthropod communities in shrubland as the fire intensity, fuel loads, and soil moisture could differ significantly between the shrubs and mottes.

The goal of this study was to test whether ground‐dwelling arthropod communities were affected by: (a) habitat type (i.e., mottes versus shrubland), (b) time since prescribe fire, and (c) the interaction between habitat type and fire. To achieve this, we examined arthropod communities across a gradient of two habitat types (i.e., mottes and shrubland at different distances from the mottes) that also included patches burned at different times since fire intervals. We hypothesized that ground‐dwelling arthropod communities would differ between mottes and the surrounding shrubland. We examined arthropods over a gradient of distance from mottes as the mobility of some arthropod groups can be relatively limited. The ground layer of closed‐canopy mottes is dominated by leaf litter and resources for detritivores, whereas the surrounding shrubland has abundant primary production that can support herbivores. We also predicted that there would be a significant interaction between habitat type and time since burning. Previous work has shown a higher abundance of invertebrates in grass and shrublands in the year after a burn, likely due to increased quantity or quality of primary production (Doxon et al., 2011; Engle et al., 2008). In contrast, in mottes there could be a decline in invertebrate abundance, especially of detritivores, if fires remove detrital resources that take time to accumulate.

## 2 METHODS

### 2.1 Study site

This study was conducted at Packsaddle Wildlife Management Area (hereafter, Packsaddle WMA) in Ellis County, Oklahoma. It is a 6,475‐ha shrub‐dominated grassland with elevations ranging from 579 to 762 m above mean sea level (Townsend et al., 2001). Soils in Packsaddle WMA consist of sandy Nobscot, Delwin, and Eda, moderately sandy Hardeman‐Likes‐Devol and Eda‐Carwile, and loamy Quinlan (Cole et al., 1966; Townsend et al., 2001; USDA‐NRCS Official Soil Series Descriptions, 2000). Dominant species of grasses include sand bluestem (*Andropogon hallii*), little bluestem (*Schizachyrium scoparium*), indiangrass (*Sorghastrum nutans*), switchgrass (*Panicum virgatum*), sand paspalum (*Paspalum stramineum*), blue grama (*Bouteloua gracilis*), hairy grama (*B. hirsuta*), and sand dropseed (*Sporobolus cryptandrus*) (Cole et al., 1966; Townsend et al., 2001). Common forbs in Packsaddle WMA include western ragweed (*Ambrosia psilostachaya*), croton (*Croton sp.),* and prairie sunflower (*Helianthus petiolaris*) (Cole et al., 1966; Townsend et al., 2001). Dominant woody vegetation includes sand shinnery oak (*Quercus harvardii*), sand sagebrush (*Artemisia filifolia*), and sand plum (*Prunus angustifolia*) (Cole et al., 1966; Townsend et al., 2001). Sand shinnery oak shrubs range between 0.25 and 1.5 m tall (Harrell & Fuhlendorf, 2002) and rarely exceed 1.5 m in height, while mottes were primarily comprised of hybrid post‐shinnery oaks and identified as a distinct patch of trees with heights averaging 2 m or greater (Peterson & Boyd, 2000).

Packsaddle WMA has been managed using prescribed burns since the 1990s with areas burned once every two to five years. The size of patch burns is highly variable and depends on weather conditions, other habitat management activities, and available personnel. Within the boundaries of Packsaddle WMA, several units are burned every two to three years, weather permitting. Many areas within Packsaddle WMA are also grazed by cattle during the growing season with a stocking rate of about seven ha per animal.

### 2.2 Sampling design

Our overall sampling design focused on comparing arthropod abundance along a gradient from mottes into open shrubland areas of different years since burn that included one year postburn, two years postburn, and unburned (i.e., at least five years postburn).

Within Packsaddle WMA, shinnery‐postoak mottes were identified in areas of known burn years using Google Earth (©Google, 2018). This study included 8 burn units. Mottes were identified as closed canopy, patches of trees. Soil types for each motte were obtained using Ecological Site Descriptions from the Natural Resource Conservation Service web soil survey application (Natural Resource Conservation Service & Web Soil Survey, 2018), and mottes were chosen within similar soil types. In addition to soil type, mottes were selected to be >100 m from the edge of a patch burn. Within each patch burn, we haphazardly selected mottes to maximize the spacing between adjacent mottes used in the study. The total sample size included 16 mottes distributed in variable fire treatments as follows: six mottes in unburned areas, five mottes in areas 2 years since burn, and five mottes in areas 1 year since burn. Mottes had an average diameter of 24.6 ± 2.2 m (range = 11–42 m). The minimum distance between mottes was approximately 100 m, and mottes were spread over a span of approximately 10 km from east to west.

Within each burn treatment, individual mottes served as a central point around which data were collected. Mottes were the unit of replication when testing for effects of burn year, and plot (i.e., a combined set of five pitfall traps in a 1 m^2^ area) was the unit of replication for testing the effects of distance from a motte. To compare mottes to open shrubland, two transects were initiated at the center of the motte and extended outward into the surrounding landscape in random directions. Square 1‐m^2^ sampling plots were placed along each transect in four locations with one plot placed at the center of the motte and three plots placed in open shrubland habitat located at 1 m, 15 m, and 50 m away from the outside edge of the motte. Therefore, eight sampling plots were placed at each motte location. Center plots were placed within the dense, shaded canopy of the motte, and were at least two meters away from the 1 m “open shrubland” plot and at least two meters away from the other, corresponding center plot. For each motte, data from the corresponding plot locations were averaged, such that for each motte there was one data point each for the center, 1, 15, and 50 m plot locations. This allowed us to observe whether mottes contained different arthropod orders relative to shrubland and whether mottes had an effect on arthropods in the surrounding landscape.

### 2.3 Vegetation measurements

To determine potential factors influencing the arthropod communities, we collected vegetation data in May and July of 2018. Vegetation sampling included woody shrub canopy cover and percent ground cover composition at each plot location. A line intercept method was used to quantify the canopy composition of woody shrubs. This method used a 20 m transect that crossed through each sampling plot within which was measured the horizontal linear length of each shrub that intercepted the line. Percent ground cover was determined using a Daubenmire frame (20 cm × 50 cm microplot marked in 10% classes) (Daubenmire, 1959). Daubenmire cover classes for grasses, forbs, bare ground, litter, and rock were recorded at three points along the vegetation transect, at each end and in the center of the study plot. Ground cover was described as a range of six cover classes including 0%–5%, 5%–25%, 25%–50%, 50%–75%, 75%–95%, and 95%–100%.

### 2.4 Arthropod collection

We used pitfall traps to sample arthropod communities once a month from May through August 2018. Each sampling plot contained five pitfall traps with one pitfall trap placed at each corner of a 1‐m^2^ plot and one pitfall trap placed in the center of the plot. Pitfall traps were 473‐ml round, plastic cups with a completely white interior, 13.3 cm deep, with a 5.7 cm bottom diameter, and a 7.6 cm top diameter. Pitfall traps were charged with 4 oz (118.3 ml) of killing solution and left active for 48 hr. The killing solution was composed of odorless and colorless propylene glycol (Pure USP, Food Grade Propylene Glycol, Momentum Fulfillment) diluted with water to 10% concentration and a few drops of clear, odorless dish soap (Seventh Generation, Inc.). This level of fluid was sufficient to submerge arthropods while avoiding the potential for the cup to overflow following rain or for arthropods to escape.

After 48 hr, all five cups at each 1‐m^2^ plot were consolidated into one sample for each sampling point along the transect. Samples were removed from the field and transferred into 70% ethanol the same day. Pitfall samples remained stored in ethanol until identified and counted in the laboratory. Following collection, traps were covered with a lid and left closed until the next month's sampling.

Following sorting of each sample, we identified arthropods to order and counted them after which samples were placed back in ethanol and stored. In some circumstances, arthropods could not be identified with complete confidence, often as a result of individuals being too damaged. These were classified as “Other.” Additionally, some orders were encountered relatively infrequently (i.e., 1% or less of the total arthropod abundance) and therefore did not represent a significant component of the arthropod community. These were also classified as “Other.”

### 2.5 Data analysis

Since each motte had two transects, the data from corresponding plots in each transect were averaged such that there was only one value per plot location (center, 1, 15, or 50 m) per motte. However, 8 of the 504 pitfall samples were disturbed, and, in these cases, we used the value for the corresponding undisturbed pitfall location rather than the average of the two locations.

The data were square root transformed to reduce the effect of highly abundant taxa while considering lesser represented orders as well. The square root transformed abundance data were visualized using a multivariate ordination procedure, nonmetric multidimensional scaling (nMDS). This analysis was done using Bray–Curtis distances in the program R using the vegan package (R package version 2.4‐5, Oksanen et al., 2017). Ordination figures allowed the evaluation of differences among plot locations and burn years in arthropod assemblage space. Plot location and burn year were individually analyzed as separate variables affecting arthropod abundance and biomass in nMDS. Tests for significance were then determined using a nonparametric multivariate statistical test, permutational multivariate analysis of variance (PERMANOVA) using the adonis function in R. Motte was used as a blocking factor using the strata option. To determine the percent dissimilarity seen in the nMDS and PERMANOVA results, we performed a SIMPER analysis using the PRIMER software (version 7, Anderson et al., 2008). The SIMPER analysis identifies which taxa of arthropods primarily contributed to the differences in community composition between treatments. For this analysis, all orders were included but we only report on those orders that contributed to the top 70% of the total dissimilarity.

Abundance of the top five most abundant arthropod orders (Collembola, Coleoptera, Hymenoptera, Acari, and Diptera) and Shannon's diversity index (Lande, 1996) were analyzed with mixed model nested ANOVAs using the software program JMP (version 14, SAS Institute, 2018). Shannon's diversity index was included as an additional metric of how the community responded to treatments. Differences in diversity among treatments were likely driven more by differences in evenness than by differences in richness, as we used arthropod order as our level of identification and most orders were recorded at most sites. These ANOVAs included motte nested within burn treatment as a random effect to include proper degrees of freedom for testing the burn treatment effect. The ANOVA models included burn year, distance from motte, and time separately and in all interactions. All abundance data were log(*x* + 1) transformed for the ANOVA analysis because log‐transformed data better approximated a normal distribution relative to other transformations. For the vegetation data, we first combined all of the variables into an overall analysis using principal components and then analyzed each vegetation variable to determine which variables contributed to the overall differences. Analysis of variance was used to test for differences in individual vegetation measures across distance from mottes and time since burns using JMP (version 14, SAS Institute, 2018). Tukey's HSD post hoc pairwise comparisons were performed in JMP (version 14, SAS Institute, 2018). Given the many statistical analyses used, we a priori set the alpha value arbitrarily to a more conservative 0.005 for evaluating statistical significance of *p*‐values to reduce the chance of type 1 errors due to multiple analyses. While we interpreted significance based on this threshold, all *p*‐values are presented in tables to allow readers to judge significance on their own.

## 3 RESULTS

We collected 206,477 arthropods from 504 pitfall traps during our study (Table A1). Overall, we collected individuals from 15 taxonomic groups of arthropods plus one group of “Other” that included all other orders that represented less than 1.0% composition, including Lepidoptera, Blattodea, Neuroptera, Isopoda, Psocoptera, Thysanoptera, and the subphylum Myriapoda.

### 3.1 Community composition

Collembola were the most numerous arthropods collected, representing 50% of all individuals, with Hymenoptera being the next most abundant, representing 30% of the total community (Table A1). All other arthropod orders represented 5% or less of the total abundance.

**FIGURE 1:**
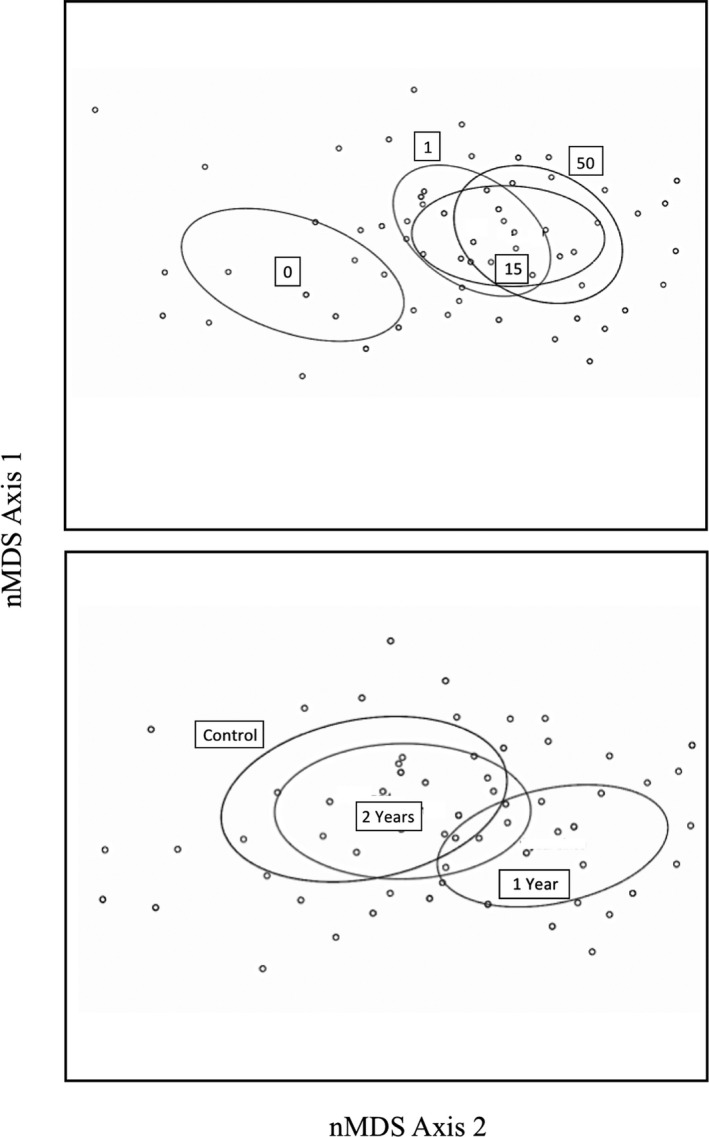
Effects of distance from a motte (0/center, 1, 15, and 50 m) and time since burning (control, 1 year, and 2 years) on total arthropod communities. Figures are nMDS ordination plots

**FIGURE A1:**
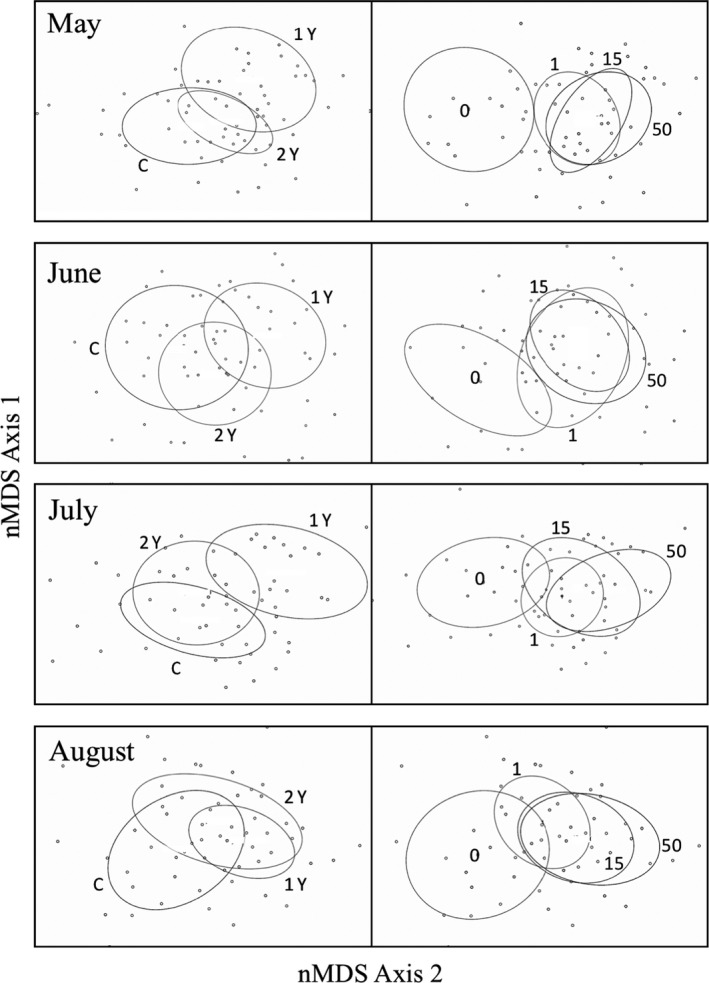
Effects of distance from a motte (0/center, 1, 15 and 50 m) and time since burning (C = Control, 1 Y = 1 Year and 2 Y = 2 Years) on arthropod communities by abundance for each of the 4 months of the study (May‐August). Figures are nMDS ordination plots

There were significant effects of burn year and distance from motte on the arthropod community, using data from all months combined and for the individual months (Table 1). August was the only month without a significant effect of burn year, and June was the only month without a significant effect of distance from a motte, using our more conservative alpha value. For burn year, arthropod communities in 1 year since burn plots appeared different from those in the control and 2 years since burn‐in nMDS plots, especially in all months combined and the months of May and July (Figure 1, Figure A1). For distance from motte, the motte location appeared more separated in space from all of the shrubland locations, which overlapped each other broadly, for all months combined and for each individual month in nMDS plots (Figure 1, Figure A1).

**Table 1:** Permutational multivariate analysis of variance (PERMANOVA) results of abundance analyses in each month separately and with all 4 months combined

Month	Source	*df*	SS	MS	Pseudo‐*F*	*P*(perm)	Permutations
May	Burn	2	0.273	0.137	5.87	0.001	999
	Location	1	0.276	0.276	11.84	0.001	999
	Burn * Location	2	0.024	0.012	0.52	0.82	999
	Residual	58	1.350	0.023			
	Total	63	1.923				
June	Burn	2	0.231	0.116	3.60	0.002	999
	Location	1	0.108	0.108	3.38	0.02	999
	Burn * Location	2	0.022	0.011	0.34	0.96	999
	Residual	58	1.864	0.032			
	Total	63	2.225				
July	Burn	2	0.274	0.137	4.42	0.002	999
	Location	1	0.223	0.223	7.18	0.002	999
	Burn * Location	2	0.018	0.009	0.294	0.96	999
	Residual	58	1.798	0.031			
	Total	63	2.312				
August	Burn	2	0.118	0.059	2.08	0.09	999
	Location	1	0.162	0.162	5.67	0.001	999
	Burn * Location	2	0.091	0.045	1.59	0.48	999
	Residual	54	1.540	0.029			
	Total	59	1.911				
All months summed	Burn	2	0.256	0.128	8.99	0.001	999
	Location	1	0.189	0.189	13.27	0.001	999
	Burn * Location	2	0.008	0.004	0.28	0.96	999
	Residual	58	0.825	0.014	0.65		
	Total	63	1.278				

Note

Burn = time since burn treatments (1 year, 2 years, and control), Location = Plot location or distance from motte (Center, 1 m, 15 m, and 50 m). Data were square root transformed. Data were collected in Packsaddle Wildlife Management Area, Oklahoma during the summer of 2018.

SIMPER results (Table A2) show that Collembola contributed the most (30%–45%) to the dissimilarity between comparisons of each burn treatment. Hymenoptera contributed between 17% and 21% of the dissimilarity across all burn years. Taxa affecting less than 11% of the dissimilarities in burn treatments observed include Diptera, Acari, and Other. Differences in community composition between plot locations were most explained by Collembola (45%–47%). Hymenoptera accounted for 13%–22% of the differences across all distances from motte. The remaining orders, Diptera, Acari, and Coleoptera, contributed less than 14% to the dissimilarities between plot locations.

### 3.2 Diversity

**FIGURE 2:**
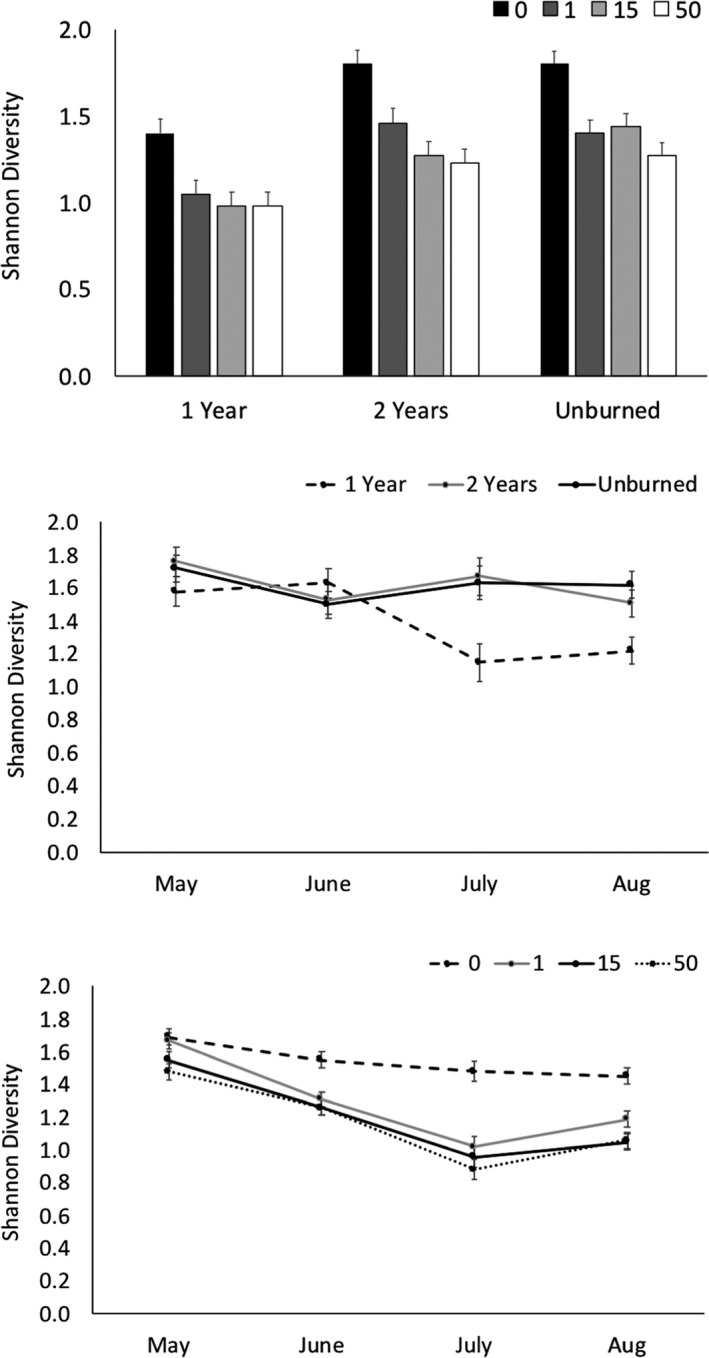
Effects of distance from a motte (0/center, 1, 15, and 50 m) and time since burning (control, 1 year, and 2 years) on the mean ± 1 *SE* Shannon diversity of (a) all arthropods summed over the course of the study, (b) arthropods by month and burn year, and (c) arthropods by month and time since burning

There were significant effects of burn year and distance from a motte on Shannon's diversity index for all arthropod data (Figure 2, Table 2). Analyzing data by month, there were significant effects of burn year, distance from motte, and time (Figure 2, Table 2). Diversity was significantly higher in mottes compared with all locations in the shrubland, and diversity was significantly lower the first year after burning compared with two or more years after burning, especially toward the last two sampling dates. There were no interactions between burn year and distance from a motte on Shannon's diversity index.

**Table 2:** Summary of the statistical analysis of Shannon diversity index on arthropod data for all months summed and for data by individual months (repeated measures)

Analysis		*df* Num	*df* Den	*F* Ratio	Prob > *F*
All months summed	Burn Year	2	42	8.14	0.001
	Plot Location	3	39	32.69	<0.0001
	Burn Year * Plot Location	6	39	0.80	0.58
Repeated measures	Burn Year	2	48	5.35	0.008
	Plot Location	3	48	29.04	<0.0001
	Burn Year * Plot Location	6	48	0.61	0.72
	Time	3	46	4.89	0.0049
	Burn Year * Time	6	92	2.69	0.02
	Plot Location * Time	9	112	2.03	0.04
	Burn Year * Plot Location * Time	18	131	1.50	0.098

### 3.3 Individual orders

**FIGURE 3:**
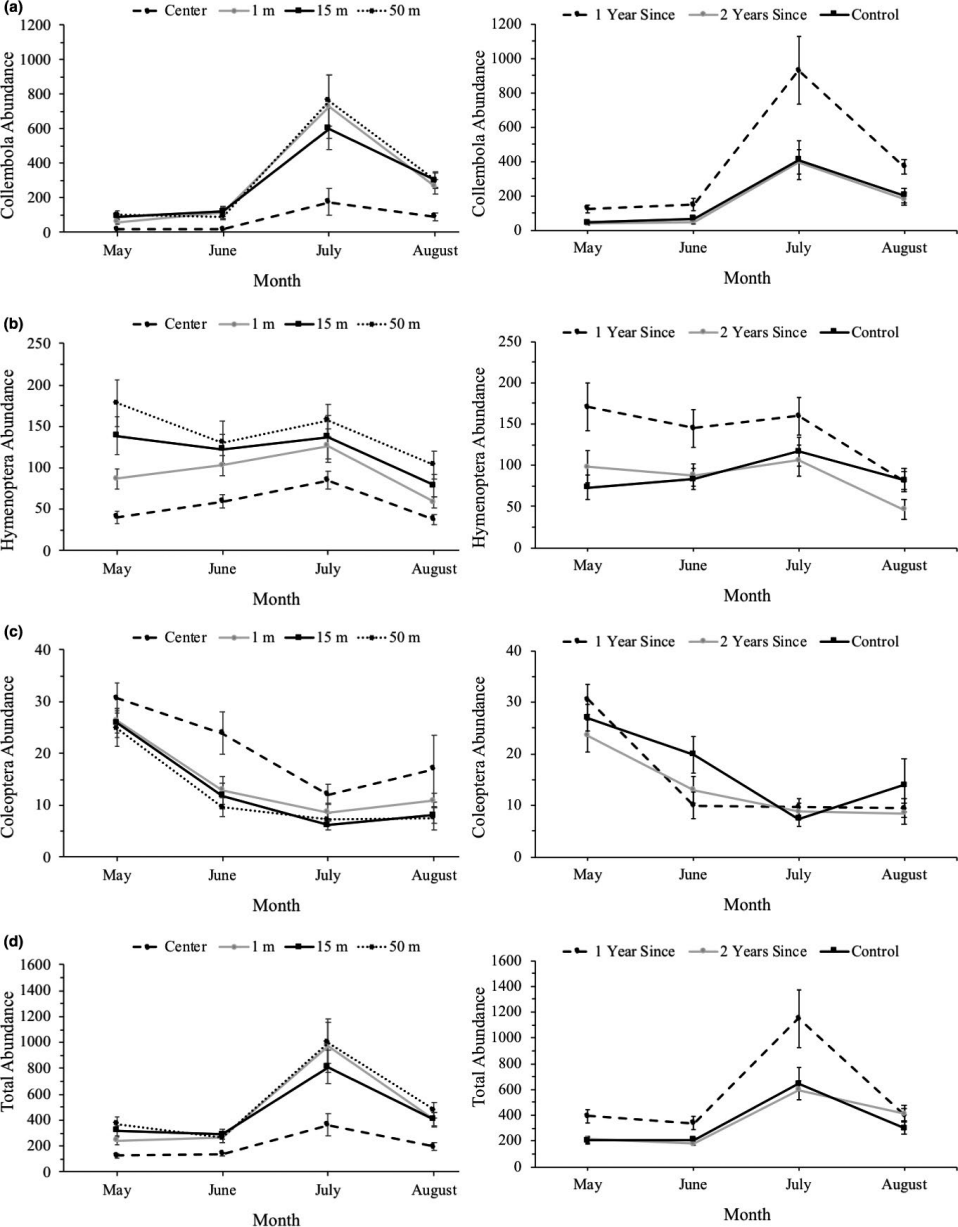
Effects of distance from a motte (0/center, 1, 15, and 50 m) and time since burning (control, 1 year, and 2 years) on the mean ± 1 *SE* abundance of (a) Collembola, (b) Hymenoptera, (c) Coleoptera, and (d) total arthropod abundance across the 4 months of the study (May–August). Collembola, Hymenoptera, and Coleoptera represent the 3 most abundant orders of arthropods collected

There were significant effects of distance from motte and time on total abundance of arthropods, summed across all 4 months, but there was no burn year by distance from motte interactions for any of the arthropod taxa. (Table A3, Figure 3). For Collembola, there were significant effects of distance from motte and time since fire on abundance. Collembola were most abundant in the 1 year since burn treatment plots relative to the other two burn treatments and they were least abundant in the center plot compared with the other distances from motte (Figure 3). For Hymenoptera, there were significant effects of distance from motte and the interaction of burn year and month sampled on abundance (Table A3). Hymenoptera were least abundant in the plots in the center of a motte relative to the other plot locations (Figure 3). For Coleoptera, there were significant effects of distance from motte and time on abundance (Table A3). Coleopterans were most abundant in the center plot relative to other plot locations (Figure 3). For Acari and Diptera, there were only significant effects of time on abundance (Table A3).

### 3.4 Vegetation

**FIGURE 4:**
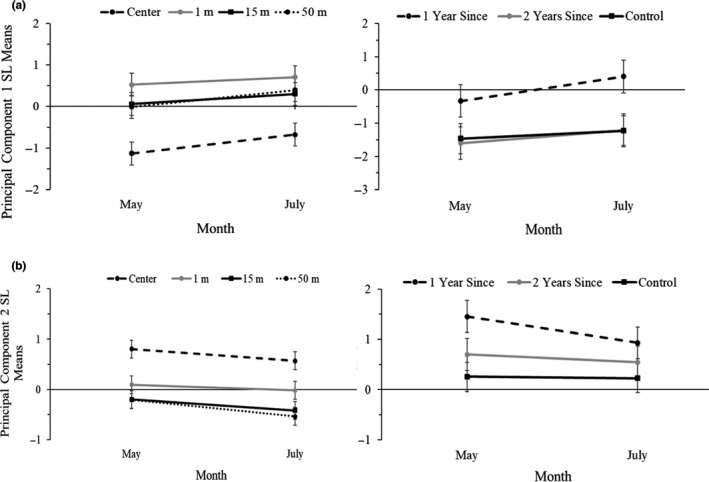
Effects of distance from a motte (0/center, 1, 15, and 50 m) and time since burning (control, 1 year, and 2 years) on the mean ± 1 *SE* vegetation for (a) principal component 1 and (b) principal component 2

For vegetation composition, the six habitat measurements were combined into two principal components. Principal component 1 had relatively high loading for shrub cover, bare ground, and litter (Table A4). Principal component 2 had relatively high loading for grass cover and forb cover. For both principal components 1 and 2, there were significant effects of distance from motte on vegetation structure (Table 3). For both principal component axes, the center plot location was different in vegetation structure relative to the other three distances from a motte (Figure 4). When analyzing the individual habitat components, there were significant effects of burn year for the percent grass composition and the percent forb composition (Table A5). Forb cover in the 1 year since burn treatment was significantly different from control burn forb composition (Table 4). Grass cover in 1 year since burn was significantly different from 2 years since burn, but neither were significantly different from the control treatment. There were significant effects of distance from motte on shrub cover, grass cover, bare ground, and litter (Table A5). Shrub cover was only significantly different between the 1 m and 15 m plot locations (Table 4). There were significant differences in grass cover, bare ground, and litter in the center plots compared with all three of the open shrubland plots. Grass cover was the only variable that showed a significant effect of time (Table A5).

**Table 3:** Summary of mixed model nested ANOVAs on principal components on vegetation measurements. ANOVAs included motte nested within burn treatment as a random effect

Component	Source	DFNum	DFDen	*F* Ratio	Prob > *F*
Prin 1	Burn	2	48.2	2.04	0.141
	Location	3	219	11.46	<0.0001
	Burn * Location	6	219	0.61	0.723
	Time	1	219	2.38	0.125
	Burn * Time	2	219	0.25	0.777
	Location * Time	3	219	0.2	0.899
	Burn * Location * Time	6	219	0.52	0.791
Prin 2	Burn	2	113.6	3.93	0.022
	Location	3	219	8.8	<0.0001
	Burn * Location	6	219	1.88	0.086
	Time	1	219	1.08	0.299
	Burn * Time	2	219	0.43	0.651
	Location * Time	3	219	0.17	0.92
	Burn * Location * Time	6	219	0.58	0.744

**Table 4:** Summary of standard least square means ± *SE* for individual mixed model nested ANOVAs of vegetation data

Vegetation measure	Burn year			Plot location			
	1 year since	2 years since	Control	Center	1 m	15 m	50 m
Percent shrub canopy	3.30 ± 0.32^a^	3.99 ± 0.32^a^	3.82 ± 0.29^a^	3.71 ± 0.18^ab^	3.45 ± 0.18^b^	4.20 ± 0.18^a^	3.99 ± 0.18^ab^
Percent grass cover	1.72 ± 0.28^a^	0.14 ± 0.28^b^	1.09 ± 0.26^ab^	0.98 ± 0.16^b^	3.04 ± 0.16^a^	3.02 ± 0.16^a^	2.96 ± 0.16^a^
Percent forb cover	2.08 ± 0.29^a^	1.02 ± 0.29^ab^	0.43 ± 0.26^b^	1.18 ± 0.16^a^	1.19 ± 0.16^a^	1.06 ± 0.16^a^	1.12 ± 0.16^a^
Percent bare ground	1.60 ± 0.37^a^	1.12 ± 0.37^a^	0.87 ± 0.34^a^	1.20 ± 0.21^b^	2.37 ± 0.21^a^	2.28 ± 0.21^a^	2.30 ± 0.21^a^
Percent litter cover	4.25 ± 0.14^a^	4.51 ± 0.14^a^	4.49 ± 0.13^a^	4.42 ± 0.08^a^	3.98 ± 0.08^b^	3.99 ± 0.08^b^	3.99 ± 0.08^b^
Percent rock	0.00 ± 0.04^a^	0.00 ± 0.04^a^	0.00 ± 0.04^a^	0.00 ± 0.02^a^	0.05 ± 0.02^a^	0.02 ± 0.02^a^	0.00 ± 0.02^a^

Note

Differences in letters across rows indicate significant differences where *α* < 0.005.

## 4 DISCUSSION

Our results do not support the hypothesis that fire and mottes interact to affect ground‐dwelling arthropod communities. Rather, our results demonstrate that fire and mottes have separate effects on arthropod communities. Mottes had a different community of arthropods compared with the surrounding shrubland. Mottes also had lower overall abundance, but a higher diversity of arthropods. In terms of fires, arthropod communities one year after burning were different from those two or more years after burning. There was no effect of burning on overall arthropod abundance, but plots that were one year since burning had significantly lower diversity compared with plots that were two or more years postburn. Hence, the findings of this study suggest that both fire and mottes can independently facilitate heterogeneity in arthropod communities, but they do not appear to interact with one another.

The transition from mottes to shrubland is fairly discrete in terms of overall vegetation structure as mottes have a closed canopy of trees >2 m tall while shrubland has much shorter vegetation. Our ground cover data support the discrete change in vegetation with mottes having lower grass, lower bare ground, and higher litter cover compared with the surrounding shrubland. The principal components analysis of vegetation also shows mottes as being distinct from shrubland plots. Shannon's diversity index, total arthropod abundance, and the abundances of Collembola and Hymenoptera also showed fairly distinct differences between motte and shrubland plots. This should be expected given that arthropod populations and communities often vary with vegetation structure, due to its influence on microclimate and food availability (Engle et al., 2008; Prather & Kaspari, 2019). Hence, the arthropod community, including the most abundant arthropods, shows a relatively discrete response to mottes versus shrubland.

Differences in arthropod communities between mottes and shrubland could be due to differences in the types of plants available as food as well as the effects of vegetation on microclimate. The shrublands in western Oklahoma can reach high temperatures during summer afternoons (Carroll et al., 2015). Mottes can serve as thermal refugia for some birds and other vertebrates (Carroll et al., 2015; Rakowski et al., 2019), and could also serve as thermal refugia for invertebrates. Our pitfall data are unable to evaluate temporal variation in use of mottes, as traps were open for two full days and nights. Opening pitfalls only for certain time periods (e.g., day or night) could help test if arthropods vary their use of mottes during the day. However, ground‐dwelling arthropods may be less likely to move in and out of mottes as they might have to move large distances to find mottes relative to other microhabitats (e.g., litter or the thick bases of clump grass or shrubs) that may provide thermal refugia. More mobile, flying arthropods (e.g., adult Orthoptera, Diptera, Hymenoptera, Hemiptera, and Lepidoptera) might be more likely to use mottes as thermal refugia.

Fire only appeared to have short‐term effects on vegetation cover and arthropod orders at our study site, as the only differences we found were between the plots burned 1 year prior and plots burned longer ago. For vegetation, there were no overall effects of prescribed burning on the vegetation principal components. But, there were some effects of fire on ground cover as plots burned 1 year prior had higher grass and forb cover compared with older burns. Fire often stimulates vegetation growth in prairie and shrubland for a year or more due to the release of nutrients when detritus is burned (Allred et al., 2011). For invertebrates, there were significant effects of fire on arthropod community composition (PERMANOVA) and diversity, with lower diversity in the plots burned 1 year prior. Of the 5 most abundant arthropod taxa, Collembola was the only one to respond to fire with higher abundance in plots 1 year since burning. Prior studies have shown that Collembola species respond differently to fire and sexually reproducing species with fast and active dispersal are more likely to recover quickly following fire (Brand, 2002; Malmström, 2012). A more detailed understanding of which species of Collembola increased in abundance and the preferred diets of these species would be needed to confirm this hypothesis. Other studies have also shown that certain arthropod taxa or communities in prairie and shrubland often recover from fire within the first year or two after a burn (Doxon et al., 2011; Swengel, 2001; Warren et al., 1987). The relatively rapid recovery of the habitat following prescribed fire in western Oklahoma may be due to the rapid growth of plants in the shrubland combined with the low intensity of the fires, due to low fuel load, which often does not kill trees in mottes or the belowground parts of shrubs in the shrubland (Malmström et al., 2008; Smit et al., 2010).

The focus of the current study was on broad responses of arthropods to habitat types (i.e., shrubland versus mottes) and time since fire. As such, our study design covered a significant area of the landscape, with 16 study plots measured over 4 months spread among patch burns on an approximately 8,000 ha wildlife management area. The breadth of this sampling necessitated a relatively coarse identification of arthropods (i.e., to order), to be able to analyze all the pitfall samples that were collected. Our results suggest that there are differences in arthropod communities between mottes and shrubland and changes in arthropod communities with time since fire. But these results do not preclude the possibility of other effects of habitat type or fire on arthropods that could be detected by more detailed taxonomic identification. For example, no change in the abundance of arthropods within a given order could be due to no change in the abundances of each family or species in that order or declines in some species with concomitant increases in other species. Furthermore, species within a single order can be diverse in their ecological roles, such as beetles that include detritivorous, herbivorous (e.g., leaf‐chewing and wood‐boring), omnivorous, and predatory species. A more detailed taxonomic study of arthropods over a shorter temporal or smaller spatial scale could provide additional insights into the role of habitat type and prescribe fire for shrubland communities.

Prescribed fire is a common management tool used to reduce woody vegetation and create habitat heterogeneity across the landscape (i.e., patch burning). Fire can have significant effects on vegetation and animal communities on its own, but is also known to interact with other factors. For example, in pyric herbivory the combination of herbivores and prescribed fire had different effects on landscapes than either alone due to the preference of herbivores for recently burned areas (Fuhlendorf & Engle, 2004; Fuhlendorf et al., 2009). In our study, we did not find support for an interaction between mottes and fire. Although this study was done in a landscape that is regularly maintained by prescribed fire, we cannot preclude the possibility that there could be interactions between fire and mottes when there are longer intervals without burning. Better understanding the potential interactions of prescribed fire with other factors in the landscape is key to using this management technique more effectively.

## CONFLICT OF INTEREST

None declared.

## AUTHOR CONTRIBUTIONS

**Shawn M. Wilder:** Conceptualization (equal); funding acquisition (equal); methodology (equal); project administration (equal); resources (lead); visualization (equal); writing—original draft preparation (supporting); writing—review & editing (equal). **Anna Butler:** Conceptualization (equal); data curation (lead); formal analysis (lead); investigation (lead); project administration (equal); visualization (equal); writing—original draft preparation (lead); writing—review & editing (equal). **Craig A. Davis:** Conceptualization (equal); funding acquisition (equal); methodology (equal); writing—review & editing (equal). **Samuel D. Fuhlendorf:** Conceptualization (equal); funding acquisition (equal); methodology (equal); writing—review & editing (equal).

## Supporting information

Appendix S1

## Data Availability

Pitfall captures, Shannon diversity, and vegetation data at Dryad: https://doi.org/10.5061/dryad.z612jm69w.

## Appendix A

**Table A1:** Total abundance of all arthropods collected in pitfall traps each month in Packsaddle Wildlife Management Area, Oklahoma during the summer of 2018

Arthropod order	Month sampled				Sum of each order
	May	June	July	August	
Coleoptera	3,412	1,767	1,076	1,306	7,561
Lepidoptera	106	56	83	79	324
Hymenoptera	14,178	12,801	16,009	8,360	51,348
Collembola	8,540	10,545	72,095	28,674	119,854
Orthoptera	668	394	910	788	2,760
Blattodea	249	296	1,280	444	2,269
Neuroptera	36	16	18	12	82
Diptera	1,321	761	4,693	1,229	8,004
Araneae	1,211	1,044	771	834	3,860
Acari	2,796	1,304	1,878	821	6,799
Isopoda	3	2	1	4	10
Hemiptera	907	347	440	534	2,228
Myriapoda	35	5	6	1	47
Psocoptera	5	233	522	106	866
Thysanoptera	237	49	53	67	406
Other	9	16	22	12	59
Sum of each month	33,713	29,636	99,857	43,271	206,477

Note

Arthropod orders included in “Other” are those that could not be identified with complete confidence, as well as some orders that were encountered relatively infrequently and therefore do not represent a significant component of the arthropod community.

**Table A2:** Results of SIMPER analyses on arthropod abundance

Comparison	Average dissimilarity	Collembola	Hymenoptera	Coleoptera	Acari	Diptera	Other	Total % explained
Control versus 2 years since	12.99	30.4	16.71	–	9.32	10.91	8	75.34
Control versus 1 year since	18.26	45.21	17.26	–	–	–	8.52	70.99
2 years since versus 1 year since	17.04	43.51	21.42	–	–	–	7.09	72.02
Center versus 1 m	21.97	47.3	13.43	–	9.42	–	–	70.15
Center versus 15 m	22.24	45.3	17.85	–	7.33	–	–	70.48
1 m versus 15 m	12.53	36.23	16.24	–	8.35	12.58	–	73.4
Center versus 50 m	25.11	45.87	20.75	–	6.45	–	–	73.07
1 m versus 50 m	12.5	32.09	21.01	–	7.21	13.44	–	73.75
15 m versus 50 m	12.08	35.71	21.55	6.62	–	9.12	–	73

Note

SIMPER analyses, similarity percentages, break down the contribution of each order to the observed dissimilarity between samples for the PERMANOVA analyses. Total % explained shows the cumulative percentage of the average dissimilarity that is explained by all orders in each row. Only orders that contributed to the top 70% of the total dissimilarity were considered. Data were square root transformed.

**Table A3:** Summary of repeated measures ANOVAs testing the effects of burn year and plot location (distance from motte) on abundance of the five most abundant orders analyzed in each month separately and with all 4 months combined

Taxa	Source	Nparm	DFNum	DFDen	*F* Ratio	Prob > *F*
Collembola	Burn	2	2	13	12.05	**0.001**
	Location	1	1	227	29.43	**<0.0001**
	Burn * Location	2	2	227	0.32	0.726
	Time	1	1	229	97.79	**<0.0001**
	Burn * Time	2	2	229	0.19	0.828
	Location * Time	1	1	227	0.37	0.543
	Burn * Location * Time	2	2	227	0.23	0.795
Hymenoptera	Burn	2	2	13	4.98	0.025
	Location	1	1	227	41.93	**<0.0001**
	Burn * Location	2	2	227	0.65	0.524
	Time	1	1	228	7.30	0.007
	Burn * Time	2	2	228	6.93	**0.001**
	Location * Time	1	1	227	2.02	0.157
	Burn * Location * Time	2	2	227	0.35	0.707
Coleoptera	Burn	2	2	11	0.36	0.706
	Location	1	1	225	14.38	**<0.001**
	Burn * Location	2	2	225	0.28	0.753
	Time	1	1	227	93.92	**<0.0001**
	Burn * Time	2	2	227	1.33	0.268
	Location * Time	1	1	225	0.85	0.357
	Burn * Location * Time	2	2	225	0.87	0.422
Acari	Burn	2	2	13	1.60	0.240
	Location	1	1	227	2.17	0.143
	Burn * Location	2	2	227	1.71	0.183
	Time	1	1	230	47.25	**<0.0001**
	Burn * Time	2	2	230	6.70	**0.002**
	Location * Time	1	1	227	1.24	0.267
	Burn * Location * Time	2	2	227	1.05	0.350
Diptera	Burn	2	2	12	0.84	0.454
	Location	1	1	226	6.69	0.010
	Burn * Location	2	2	226	0.49	0.614
	Time	1	1	227	10.74	**0.001**
	Burn * Time	2	2	227	0.31	0.732
	Location * Time	1	1	226	3.05	0.082
	Burn * Location * Time	2	2	226	0.58	0.561
Total	Burn	2	2	158	1.40	0.250
	Location	3	3	191	15.45	**<0.0001**
	Burn * Location	6	6	191	0.15	0.988
	Time	3	3	191	14.33	**<0.0001**
	Burn * Time	6	6	191	1.37	0.227
	Location * Time	9	9	191	1.02	0.427
	Burn * Location * Time	18	18	191	0.69	0.821

Note

Burn = time since burn treatments (1‐year, 2 years, and control), Plot = Pot location or distance from motte (Center, 1 m, 15 m, and 50 m). ANOVAs included motte nested within burn treatment as a random effect. Data were log(*x* + 1) transformed. *p*‐values <.005 in bold. Data were collected at Packsaddle Wildlife Management Area, Oklahoma during the summer of 2018.

**Table A4:** Principal Component Analysis eigenvectors showing loading of vegetation measures on both principal components

Vegetation measure	Principal components	
	Prin 1	Prin 2
Percent shrub canopy	−0.40851	−0.25383
Percent grass	0.47695	−0.5195
Percent forb	0.18698	0.77053
Percent bare ground	0.38271	0.22167
Percent litter	−0.63905	0.11299
Percent rock	0.12579	−0.10031

**Table A5:** Summary of mixed model nested ANOVAs on principal components on vegetation measurements

Measure	Source	DFNum	DFDen	*F* Ratio	Prob > *F*
Percent shrub canopy	Burn	2	42.4	1.25	0.298
	Location	3	219	6.30	**<0.001**
	Burn * Location	6	219	0.81	0.565
	Time	1	219	0.04	0.834
	Burn * Time	2	219	1.97	0.143
	Location * Time	3	219	0.06	0.979
	Burn * Location * Time	6	219	0.61	0.720
Percent grass cover	Burn	2	78.6	7.91	**0.001**
	Location	3	219	57.48	**<0.0001**
	Burn * Location	6	219	1.84	0.092
	Time	1	219	10.78	**0.001**
	Burn * Time	2	219	3.73	0.026
	Location * Time	3	219	1.63	0.183
	Burn * Location * Time	6	219	1.10	0.364
Percent forb cover	Burn	2	110.1	8.96	**<0.001**
	Location	3	219	0.17	0.918
	Burn * Location	6	219	2.90	0.010
	Time	1	219	0.41	0.524
	Burn * Time	2	219	0.06	0.944
	Location * Time	3	219	0.36	0.781
	Burn * Location * Time	6	219	0.94	0.469
Percent bare ground cover	Burn	2	92.2	1.07	0.347
	Location	3	219	9.75	**<0.0001**
	Burn * Location	6	219	1.55	0.163
	Time	1	219	0.84	0.360
	Burn * Time	2	219	1.11	0.333
	Location * Time	3	219	0.43	0.728
	Burn * Location * Time	6	219	0.87	0.514
Percent litter cover	Burn	2	85.8	1.07	0.347
	Location	3	219	9.59	**<0.0001**
	Burn * Location	6	219	0.26	0.953
	Time	1	219	2.17	0.142
	Burn * Time	2	219	0.81	0.446
	Location * Time	3	219	0.06	0.980
	Burn * Location * Time	6	219	1.12	0.353
Percent rock cover	Burn	2	53	0.00	1.000
	Location	3	219	2.22	0.086
	Burn * Location	6	219	0.80	0.572
	Time	1	219	0.66	0.418
	Burn * Time	2	219	0.70	0.497
	Location * Time	3	219	1.25	0.293
	Burn * Location * Time	6	219	0.67	0.673

Note

ANOVAs included motte nested within burn treatment as a random effect. Data were log (*x* + 1) transformed. *p*‐values <.005 in bold.

## References

[ece37063-bib-0001] Allred, B. W. , Fuhlendorf, S. D. , Engle, D. M. , & Elmore, R. D. (2011). Ungulate preference for burned patches reveals strength of fire‐grazing interaction. Ecology and Evolution, 1, 132–144. 10.1002/ece3.12 22393490 PMC3287302

[ece37063-bib-0002] Anderson, M. J. , Gorley, R. N. , & Clarke, K. R. (2008). PERMANOVA+ for PRIMER: Guide to software and statistical methods. PRIMER‐E.

[ece37063-bib-0003] Brand, R. H. (2002). The effects of prescribed burning on epigeic springtails (Insecta: Collembola) of woodland litter. American Midland Naturalist, 148, 383–393.

[ece37063-bib-0004] Carroll, J. M. , Davis, C. A. , Elmore, R. D. , Fuhlendorf, S. D. , & Thacker, E. T. (2015). Thermal patterns constrain diurnal behavior of a ground‐dwelling bird. Ecosphere, 6(11), 1–15. 10.1890/ES15-00163.1

[ece37063-bib-0005] Cole, E. L. , Conradi, A. J. , & Rhoads, C. E. (1966). Soil survey, Ellis County, Oklahoma.

[ece37063-bib-0006] Daubenmire, R. F. (1959). Canopy coverage method of vegetation analysis. Northwest Science, 33, 39–64.

[ece37063-bib-0007] Deutsch, C. A. , Tewksbury, J. J. , Huey, R. B. , Sheldon, K. S. , Ghalambor, C. K. , Haak, D. C. , & Martin, P. R. (2008). Impacts of climate warming on terrestrial ectotherms across latitude. Proceedings of the National Academy of Sciences, 105(18), 6668–6672. 10.1073/pnas.0709472105 PMC2373333 18458348

[ece37063-bib-0008] Doxon, E. D. , Davis, C. A. , Fuhlendorf, S. D. , & Winter, S. L. (2011). Aboveground macroinvertebrate diversity and abundance in sand sagebrush prairie managed with use of pyric herbivory. Rangeland Ecology & Management, 64, 394–403.

[ece37063-bib-0009] Engle, D. M. , Fuhlendorf, S. D. , Roper, A. , & Leslie, D. M. Jr (2008). Invertebrate community response to a shifting mosaic of habitat. Rangeland Ecology & Management, 61(1), 55–62. 10.2111/06-149R2.1

[ece37063-bib-0010] Fuhlendorf, S. D. , & Engle, D. M. (2004). Application of the fire–grazing interaction to restore a shifting mosaic on tallgrass prairie. Journal of Applied Ecology, 41(4), 604–614. 10.1111/j.0021-8901.2004.00937.x

[ece37063-bib-0011] Fuhlendorf, S. D. , Engle, D. M. , Kerby, J. , & Hamilton, R. (2009). Pyric herbivory: Rewilding landscapes through recoupling of fire and grazing. Conservation Biology, 23, 588–598. 19183203 10.1111/j.1523-1739.2008.01139.x

[ece37063-bib-0012] Fuhlendorf, S. D. , Harrell, W. C. , Engle, D. M. , Hamilton, R. G. , Davis, C. A. , & Leslie, D. M. Jr (2006). Should heterogeneity be the basis for conservation? Grassland bird response to fire and grazing. Ecological Applications, 16(5), 1706–1716. 17069365 10.1890/1051-0761(2006)016[1706:shbtbf]2.0.co;2

[ece37063-bib-0013] Gillespie, M. A. , Alfredsson, M. , Barrio, I. C. , Bowden, J. J. , Convey, P. , Culler, L. E. , Coulson, S. J. , Krogh, P. H. , Koltz, A. M. , Koponen, S. , Loboda, S. , Marusik, Y. , Sandström, J. P. , Sikes, D. S. , & Høye, T. T. (2020). Status and trends of terrestrial arthropod abundance and diversity in the North Atlantic region of the Arctic. Ambio, 49, 718–731. 30879270 10.1007/s13280-019-01162-5 PMC6989714

[ece37063-bib-0014] Hallmann, C. A. , Sorg, M. , Jongejans, E. , Siepel, H. , Hofland, N. , Schwan, H. , Stenmans, W. , Müller, A. , Sumser, S. , Hörren, T. , Goulson, D. , & de Kroon, H. (2017). More than 75 percent decline over 27 years in total flying insect biomass in protected areas. PLoS One, 12(10), e0185809 10.1371/journal.pone.0185809 29045418 PMC5646769

[ece37063-bib-0015] Harrell, W. C. , & Fuhlendorf, S. D. (2002). Evaluation of habitat structural measures in a shrubland community. Rangeland Ecology & Management/Journal of Range Management Archives, 55(5), 488–493. 10.2307/4003227

[ece37063-bib-0016] Klink, R. V. , Bowler, D. E. , Gongalsky, K. B. , Swengel, A. B. , Gentile, A. , & Chase, J. M. (2020). Meta‐analysis reveals declines in terrestrial but increases in freshwater insect abundances. Science, 368, 417–420. 32327596 10.1126/science.aax9931

[ece37063-bib-0017] Lande, R. (1996). Statistics and partitioning of species diversity, and similarity among multiple communities. Oikos, 76, 5–13. 10.2307/3545743

[ece37063-bib-0018] Lister, B. C. , & Garcia, A. (2018). Climate‐driven declines in arthropod abundance restructure a rainforest food web. Proceedings of the National Academy of Sciences, 115(44), E10397–E10406. 10.1073/pnas.1722477115 PMC6217376 30322922

[ece37063-bib-0019] Malmström, A. (2012). Life‐history traits predict recovery patterns in Collembola species after fire: A 10 year study. Applied Soil Ecology, 56, 35–42. 10.1016/j.apsoil.2012.02.007

[ece37063-bib-0020] Malmström, A. , Persson, T. , & Ahlström, K. (2008). Effects of fire intensity on survival and recovery of soil microarthropods after a clearcut burning. Canadian Journal of Forest Research, 38(9), 2465–2475. 10.1139/X08-094

[ece37063-bib-0021] May, M. L. (1979). Insect thermoregulation. Annual Review of Entomology, 24(1), 313–349. 10.1146/annurev.en.24.010179.001525

[ece37063-bib-0022] Natural Resources Conservation Service (n.d.). Web soil survey. United States Department of Agriculture Retrieved from https://websoilsurvey.nrcs.usda.gov/app/WebSoilSurvey.aspx

[ece37063-bib-0023] Oksanen, J. , Blanchet, F. G. , Friendly, M. , Kindt, R. , Legendre, P. , McGlinn, D. , Minchin, P. R. , O'Hara, R. B. , Simpson, G. L. , Solymos, P. , Stevens, M. H. H. , Szoecs, E. , & Wagner, H. (2017). vegan: Community ecology package. R package version 2.4‐5. Retrieved from https://CRAN.R‐project.org/package=vegan

[ece37063-bib-0024] Peterson, R. , & Boyd, C. S. (2000). Ecology and management of sand shinnery communities: A literature review. Gen. Tech. Rep. RMRS‐GTR‐16 (44 p., 16). : US Department of Agriculture, Forest Service, Rocky Mountain Research Station.

[ece37063-bib-0025] Prather, R. M. , & Kaspari, M. (2019). Plants regulate grassland arthropod communities through biomass, quality and habitat heterogeneity. Ecosphere, 10, e02909 10.1002/ecs2.2909

[ece37063-bib-0026] Rakowski, A. E. , Elmore, R. D. , Davis, C. A. , Fuhlendorf, S. D. , & Carroll, J. M. (2019). Thermal refuge affects space use and movement of a large‐bodied galliform. Journal of Thermal Biology, 80, 37–44. 10.1016/j.jtherbio.2018.12.024 30784486

[ece37063-bib-0027] Reed, C. C. (1997). Responses of prairie insects and other arthropods to prescription burns. Natural Areas Journal, 17, 380–385.

[ece37063-bib-0028] Robertson, R. , Kuhnert, C. , & Dawson, J. (1996). Thermal avoidance during flight in the locust *Locusta migratoria* . Journal of Experimental Biology, 199(6), 1383–1393. 10.1242/jeb.199.6.1383 9319276

[ece37063-bib-0029] Sánchez‐Bayo, F. , & Wyckhuys, K. A. (2019). Worldwide decline of the entomofauna: A review of its drivers. Biological Conservation, 232, 8–27. 10.1016/j.biocon.2019.01.020

[ece37063-bib-0030] SAS Institute Inc (2018). JMP® Version 14. Cary, NC.

[ece37063-bib-0031] Seastedt, T. R. , & Crossley, D. A. (1984). The influence of arthropods on ecosystems. BioScience, 34, 157–161.

[ece37063-bib-0032] Smit, I. P. J. , Asner, G. P. , Govender, N. , Kennedy‐Bowdoin, T. , Knapp, D. E. , & Jacobson, J. (2010). Effects of fire on woody vegetation structure in African savanna. Ecological Applications, 20, 1865–1875. 10.1890/09-0929.1 21049875

[ece37063-bib-0033] Swengel, A. B. (2001). A literature review of insect responses to fire, compared to other conservation managements of open habitat. Biodiversity and Conservation, 10, 1141–1169.

[ece37063-bib-0034] Townsend, D. E. , Masters, R. E. , Lochmiller, R. L. , Leslie, D. M. Jr , Demaso, S. J. , & Peoples, A. D. (2001). Characteristics of nest sites of northern bobwhites in western Oklahoma. Journal of Range Management, 260–264. 10.2307/4003244

[ece37063-bib-0035] United States Department of Agriculture‐Natural Resource Conservation Service (2000). Official soil series descriptions. Retrieved from http://www.statlab.iastate.edu/cgi

[ece37063-bib-0036] Wagner, D. L. (2020). Insect declines in the Anthropocene. Annual Review of Entomology, 65, 457–480. 10.1146/annurev-ento-011019-025151 31610138

[ece37063-bib-0037] Warren, S. D. , Scifres, C. J. , & Teel, P. D. (1987). Responses of grassland arthropods to burning: A review. Agriculture, Ecosystems and Environment, 19, 105–130.

[ece37063-bib-0038] Weideman, V. E. , & Penfound, W. T. (1960). A preliminary study of the shinnery in Oklahoma. Southwestern Naturalist, 5, 117–122. 10.2307/3669506

