# Supplementary material for: Effects of fire on ground‐dwelling arthropods in a shrub‐dominated grassland
Source: Ecol Evol. 2020 Dec 7;11(1):427–42. doi: 10.1002/ece3.7063 (PMC7790617; doi:10.1002/ece3.7063)

**Appendix**

**Table A1**. Total abundance of all arthropods collected in pitfall traps each month in Packsaddle Wildlife Management Area, Oklahoma during the summer of 2018. Arthropod orders included in “Other” are those that could not be identified with complete confidence, as well as some orders that were encountered relatively infrequently and therefore do not represent a significant component of the arthropod community.

**Table A2**. Results of SIMPER analyses on arthropod abundance. SIMPER analyses, similarity percentages, break down the contribution of each order to the observed dissimilarity between samples for the PERMANOVA analyses. Total % explained shows the cumulative percentage of the average dissimilarity that is explained by all orders in each row. Only orders that contributed to the top 70% of the total dissimilarity were considered. Data were square root transformed.

**Table A3**. Summary of repeated measures ANOVAs testing the effects of burn year and plot location (distance from motte) on abundance of the five most abundant orders analyzed in each month separately and with all four months combined. Burn = time since burn treatments (1-year, 2 years, and control), Plot = Pot location or distance from motte (Center, 1m, 15m, and 50m). ANOVAs included motte nested within burn treatment as a random effect. Data were log(x+1) transformed. P-values <0.005 in bold. Data were collected at Packsaddle Wildlife Management Area, Oklahoma during the summer of 2018.

**Table A4**. Principal Component Analysis eigenvectors showing loading of vegetation measures on both principal components.

|  | **Principal Components** | |
| --- | --- | --- |
| **Vegetation Measure** | **Prin 1** | **Prin 2** |
| Percent Shrub Canopy | -0.40851 | -0.25383 |
| Percent Grass | 0.47695 | -0.5195 |
| Percent Forb | 0.18698 | 0.77053 |
| Percent Bare Ground | 0.38271 | 0.22167 |
| Percent Litter | -0.63905 | 0.11299 |
| Percent Rock | 0.12579 | -0.10031 |

**Table A5**. Summary of mixed model nested ANOVAs on principal components on vegetation measurements. ANOVAs included motte nested within burn treatment as a random effect. Data were log (x+1) transformed. P-values <0.005 in bold.

**Figure A1.** Effects of distance from a motte (0/center, 1, 15 and 50 m) and time since burning (C = Control, 1Y = 1 Year and 2Y = 2 Years) on arthropod communities by abundance for each of the 4 months of the study (May - August). Figures are nMDS ordination plots.


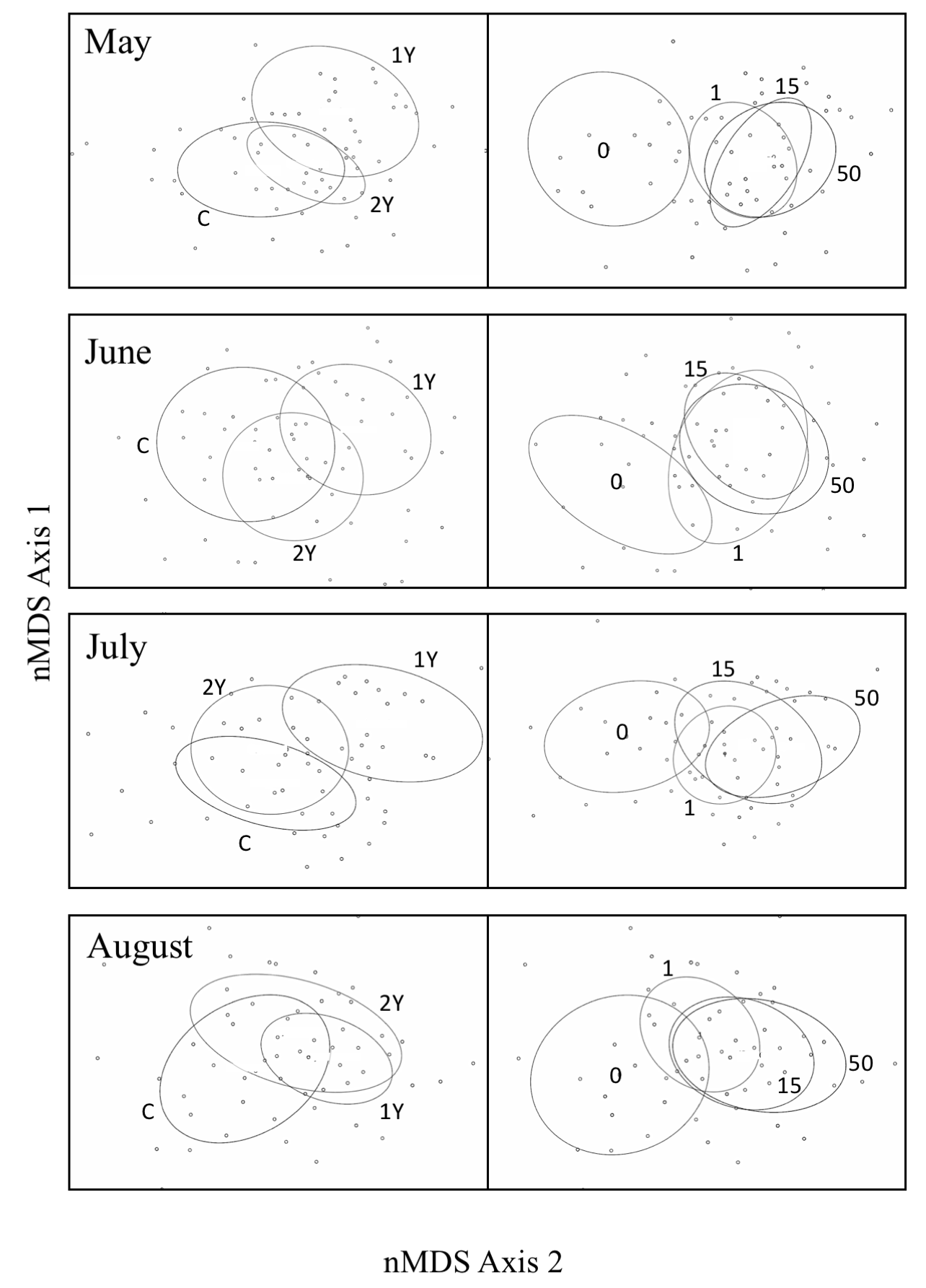

Supplement: Supplementary file 1 — Appendix S1 [file ECE3-11-427-s001.docx]
